# Safety of geriatric patients undergoing endobronchial ultrasound-guided transbronchial needle aspiration with deep sedation: a retrospective study

**DOI:** 10.1186/s12871-023-02241-7

**Published:** 2023-08-16

**Authors:** Mehtap Tunç, Hilal Sazak, Ayperi Öztürk, Aydın Yılmaz, Ali Alagöz

**Affiliations:** 1 Department of Anesthesiology and Reanimation, University of Health Sciences, Atatürk Sanatoryum Training and Research Hospital, Ankara, Turkey; 2 Department of Interventional Pulmonology, University of Health Sciences, Atatürk Sanatorium Training and Research Hospital, Ankara, Turkey

**Keywords:** Sedation, Sedative agents, Endobronchial ultrasound guided transbronchial needle aspiration, Geriatric population, Elderly, Safety, Complication

## Abstract

**Background:**

Endobronchial ultrasound-guided transbronchial needle aspiration (EBUS-TBNA) can be performed in a wide range, from minimal sedation to general anesthesia. Advanced age increases perioperative risks related to anesthesia and is also associated with many pathological processes that further increase morbidity and mortality. The ideal sedation protocol for EBUS-TBNA has yet to be determined in geriatric patients. Deep sedation (DS) may increase the safety and performance of the procedure. There are limited studies evaluating the effectiveness and safety of EBUS-TBNA under DS in elderly patients.

**Methods:**

280 patients who underwent EBUS-TBNA under DS were included in this retrospective study. 156 patients aged 65 years and over (Group 1) and 124 patients under 45 (Group 2) were compared. Demographic data, comorbidities, pulmonary function tests (PFTs), hemodynamic measurements, and peripheral oxygen saturation (SpO_2_) before the procedure were evaluated. In addition, the duration of the EBUS-TBNA procedure, sedation agents and dosages, recovery time, and complications related to the procedure in the 24 h and applied medications and treatments were recorded.

**Results:**

There was no difference in body mass index, EBUS-TBNA procedure duration, and recovery time between geriatric and young patients(p > 0.05). The proportion of female patients, pre-anesthesia SpO_2_, and PFTs were found to be significantly lower in geriatric patients(p < 0.05). ASA classification, frequency of comorbidities, and initial mean arterial pressure were found to be significantly higher in the geriatric group(p < 0.05). The propofol-ketamine combination was the most preferred sedative in both groups. The dose of propofol used in the regimen in which propofol was administered alone was found to be lower in the elderly group (p < 0.05). The increase in the HR was significant in Group 2 in the T4 and T5 periods with respect to T1 when the differences were compared (p < 0.05). As a complication, the frequency of high blood pressure during the procedure was higher in the elderly group (p < 0.05).

**Conclusions:**

The EBUS-TBNA procedure performed under DS was safe in elderly and young patients. Our study showed that the procedure and recovery times were similar in the elderly and young groups. The incidence of temporary high blood pressure during the procedure was higher in the elderly patients. The other complication rates during the procedure were similar in groups. Decreased propofol dose in the regimen using propofol alone has shown us that anesthetists are more sensitive to the administration of sedative agents in geriatric patients, taking into account comorbidities and drug interactions.

## Background

Endobronchial ultrasound-guided transbronchial needle aspiration (EBUS-TBNA), a minimally invasive technique, is commonly used in interventional pulmonology. It is used to evaluate mediastinal and hilar lymph nodes and effectively determine the stage of lung cancer [1–6]. It is also recommended to diagnose suspected cases of lymphoma, sarcoidosis, and tuberculosis [2, 5–8].

EBUS-TBNA is performed using a specialized ultrasonic bronchoscope with a larger diameter than a fiberoptic bronchoscope(FOB) [7]. While FOB can be performed routinely only under mild sedation or local anesthesia, EBUS-TBNA can be performed in a wide range from minimal sedation to general anesthesia [5–13]. Ideal sedation for EBUS-TBNA is aimed to increase diagnostic efficiency, prevent complications, and improve patient comfort by providing adequate procedural tolerance [3, 7, 8, 13–16]. The choice of sedation type varies according to institutional resources and approaches. Each institution usually determines its algorithms [7, 8, 10, 13, 17, 18].

In the expert panel on EBUS-TBNA, sedation was defined as a change in the level of consciousness, and it was divided into four categories: anxiolysis (middle sedation), conscious sedation (moderate sedation), deep sedation (DS), and general anesthesia. They suggest that conscious sedation and DS are acceptable approaches for EBUS-TBNA, with a recommendation grade of 2 C [3].

Most lung cancers occur in people over the age of 65. Accurate staging is crucial in elderly patients in determining prognosis and treatment [2]. Advanced age increases perioperative risks related to anesthesia and is also associated with many pathological processes that further increase morbidity and mortality [19]. With advancing age, the decrease in the functional capacities of the organs and the accompanying diseases contribute more to physiological regression. As a result, pharmacokinetic and pharmacodynamic approaches to anesthesia management change with age. Additionally, numerous drugs increase the risk of drug interactions and side effects [20].

Previous studies have shown that diagnostic efficiency and complication rates can be safely achieved with mild or moderate sedation in elderly patients undergoing EBUS-TBNA [9, 21–23]. However, another study reported that 1.06% of outpatients who underwent EBUS-TBNA under moderate sedation or deep sedation-general anesthesia were hospitalized or referred to the intensive care unit. The patient’s age over 70, deep sedation or general anesthesia, and hospitalization were identified as risk factors for escalation of care [17].

The ideal sedation protocol for EBUS-TBNA has yet to be determined. There are limited studies evaluating the effectiveness and safety of EBUS-TBNA under sedation in elderly patients. DS may increase the safety and performance of the procedure [10, 12, 13].

In previous studies, unlike our study, patients aged 65, 70, and 80 years and older were compared to all patients under these ages. In this retrospective study, we aimed to compare the sedation characteristics, hemodynamic data, and complications of patients aged 65 and over and aged 45 and under who underwent EBUS-TBNA with DS.

## Materials and methods

This retrospective study was conducted after obtaining ethical committee approval from the Ankara Atatürk SanatoriumTraining and Research Hospital (2012-KAEK-15/2584). Medical records of patients with American Society of Anesthesiologists (ASA) I-IV physical conditions, who received informed consent, and who underwent EBUS-TBNA with deep sedation in one year were retrospectively reviewed. Patients aged 45–65(n = 362) who underwent EBUS-TBNA were excluded from the study. A total of 280 patients were evaluated. Elderly patients aged 65 and over (Group 1, n = 156) were compared with young patients aged 45 and under (Group 2, n = 124).

The age, body mass index (BMI), gender, hemodynamic measurements, peripheral oxygen saturation (SpO_2_) during the procedure, results of the pulmonary function tests (PFTs), any accompanying comorbidities (such as chronic diseases like diabetes mellitus (DM), hypertension (HT), coronary artery disease (CAD), chronic obstructive pulmonary disease (COPD), and medications previously used by the patients were evaluated. In addition, the duration of the EBUS-TBNA procedure, sedation agents and dosages used during induction and maintenance, recovery time, and complications recorded during the 24 h during or after the procedure (bleeding, chest pain, pneumothorax, pneumomediastinum, hypertension, hypotension, bradycardia, tachycardia, arrhythmia, allergic rash, desaturation, respiratory depression, excessive sedation, need for ambu or intubation/cardiac arrest), and applied medications and treatments were recorded. Before sedation (T1), after induction (T2), after the ultrasonic bronchoscope passed the vocal cords (T3), and every 3 min during the procedure (T4, T5), systolic arterial pressure, diastolic arterial pressure, mean blood pressure (MBP), heart rate (HR), and SpO_2_ were recorded.

In our clinic, sedation for the EBUS-TBNA procedure was routinely administered in operating room conditions, according to the Ramsey Sedation Scale (RSS), with patients sedated to levels 4–5. According to this score: 1, the patient is anxious and agitated; 2, the patient is cooperative and orientated; 3, the patient responds to verbal stimulation only; 4, the patient is asleep and rapidly responses to light stimulation or loud auditory stimulus; 5, the patient is asleep but slowly responses to light stimulation or loud auditory stimulus; 6, the patient does not response to any stimulation.

All patients receiving sedation had an electrocardiogram, MBP, HR, and SpO_2_ monitoring in the operating room. Before the procedure, all patients were given 2% lidocaine spray locally to the oropharynx, and a large bore intravenous catheter was placed. During the procedure, 4 L/min of nasal oxygen was administered.

Sedation for EBUS-TBNA is administered in different protocols under the management of various anesthesia specialists. However, according to the protocols accepted by our clinic, it is applied in the form of propofol-midazolam, propofol-ketamine, propofol-ketamine-midazolam combinations, and only propofol. In our clinic, propofol and ketamine are administered at 0.25-0.5 mg/kg and midazolam at 0.03-0.05 mg/kg for induction in combined groups commonly. Propofol alone is implemented at 0.5 mg/kg for induction. In maintenance, all sedative agents are administered at a dose of approximately 0.25 mg/kg. However, in maintenance, dose adjustment is made by individual titration according to the patient’s sedation level, general condition, and anesthetist’s preference. Propofol 10 mg/cc, ketamine 10 mg/cc, and midazolam 1 mg/cc were drawn into syringes and administered. When an RSS level of 4 was reached, the was passed through the vocal cords to start the procedure. When the RSS fell below four during the procedure, a maintenance sedation agent was administered.

Biopsies were taken with the EBUS-TBNA procedure after bronchoscopic evaluation. All EBUS-TBNA procedures were performed by the same bronchoscopy team. During the procedure, if there was a 20% increase in systolic blood pressure (SBP) or if nitroglycerin was needed, it was recorded as “high blood pressure”; if there was a 20% decrease in SBP or if ephedrine was needed, it was recorded as “low blood pressure.“ If there was an allergic rash and antihistamines or steroids were given, it was considered an allergic reaction.

If SpO_2_ was below 90% during sedation, it was recorded as desaturation, and the oxygen flow was increased to 6–10 L/min, and a jaw thrust maneuver was performed. If there was no response (SpO_2_ did not recover within two minutes and fell below 88%), it was recorded as respiratory depression and ambu and/or endotracheal intubation was performed. At the end of the procedure, the time passed between the removal of the bronchoscope from the vocal cords and having the modified Aldrete 9 was determined as the recovery time. After recovery, patients were sent transferred to the ward.

All of the data obtained from the medical records were analyzed, and a comparison was made between the patient groups 65 years and older and those 45 years and younger. These two groups were analyzed in terms of the sedation agents and doses preferred, side effects observed during the procedure, administered drugs, procedure time, and recovery time.

Statistical analysis of the data was performed using the SPSS package program (Version 22.0, SPSS Inc, Chicago, IL, USA). Descriptive statistics were reported as mean ± standard deviation (SD) or median (minimum-maximum) according to the normal distribution of continuous variables. Descriptive statistics of categorical data were given as numbers and percentages. The normality distribution of the data was evaluated using Kolmogorov-Smirnov and Shapiro-Wilk tests. The Mann-Whitney U test was used to compare the non-normal numerical data, and the Student-t test was used to compare the normal data. A p-value of < 0.05 was considered statistically significant.

## Results

In our study, during a one-year study period in which EBUS-TBNA was performed with DS, a total of 280 patients were compared in terms of sedation characteristics, procedure duration, recovery time, hemodynamic data, and complications between 156 patients aged 65 and older (Group 1) and 124 patients aged 45 and younger (Group 2).

There was no statistically significant difference in terms of BMI between Group 1 and Group 2 (p > 0.05) (Table 1). In Group 1, the proportion of female patients, pre-anesthesia SpO_2_ values, FEV1, FVC, and FEV1/FVC ratios were found to be statistically significantly lower than in Group 2. In Group 1, ASA classification and comorbidities were found to be statistically significantly higher than in Group 2 (p < 0.05) (Table 1).

**Table 1:** Patient Characteristics

Parameters	Group 1 ≥ 65 Age (n:156)	Group 2 ≤ 45 Age (n:124)	p-value
**Gender**			0.007
Male	121 (%77.6)	78 (%62.9)	
Female	35 (%22.4)	46 (%37.1)	
**BMI (kg/m** ^**2**^ **)**	25.9 ± 4.6	25.9 ± 5.1	0.999
**ASA Classification**	3 (1–4)	2 (1–4)	**< 0.001**
**Comorbidities**	131 (%84.0)	42 (%33.9)	**< 0.001**
DM	33 (%21.2)	4 (%3.2)	**< 0.001**
HT	68 (%43.6)	6 (%4.8)	**< 0.001**
CAD	32 (%20.5)	3 (%2.4)	**< 0.001**
COPD	25 (%16.0)	1 (%0.8)	**< 0.001**
**FEV**_**1**_ **%**	74 (23–169)	88 (39–133)	**< 0.001**
**FVC %**	74 (22–147)	89 (40–126)	**< 0.001**
**FEV**_**1**_ **/FVC %**	76 (47–100)	82 (68–99)	**< 0.001**
**Initial hemodynamics**			
MBP (mmHg)	93.1 ± 13.0	88.4 ± 9.9	**< 0.001**
HR (beat/min)	81.9 ± 13.3	84.9 ± 12.7	0.064
SpO_2_ (%)	96.1 ± 2.3	96.7 ± 1.8	**0.027**

BMI: Body mass index. ASA:American Society of Anesthesiologists. DM: Diabetes mellitus. HT: Hypertension. CAD: Coronary artery disease. COPD: Chronic obstructive pulmonary disease. FEV1: Forced expiratory volume at 1 s. FVC: Forced vital capacity. MBP: Mean blood pressure. HR: Hearth rate. SpO_2_: Peripheral oxygen saturation

Data are expressed as n (%), mean ± SD or median (min-max) a: p < 0.05: Significant statistically difference among group

There was no statistically significant difference between the groups in terms of EBUS-TBNA procedure duration and recovery time (p > 0.05) (Table 2). The mean EBUS-TBNA duration in Groups 1 and 2 was found to be 16.24 ± 5.18 min / 16.78 ± 4.95 min, and the mean recovery times were 15.11 ± 3.79 min / 15.91 ± 3.83 min, respectively.

**Table 2:** Clinical Features of Patients according to Age Groups

Parameters	Group 1 ≥ 65 Age (n:156)	Group 2 ≤ 45 Age (n:124)	p-value
**EBUS-TBNA** **Duration(min)**	15 (10–35)	15 (10–35)	0.233
**Recovery Time (min)**	16 (9–28)	16 (9–26)	0.117
**Frequency of Anesthetic Agent Use**			**0.012**
Propofol-Midazolam	46 (%29.5)	26 (%21.0)	
Propofol-Ketamine	59 (%37.8)	61 (%49.2)	
Propofol-Ketamine-Midazolam	26 (%16.7)	29 (%23.4)	
Propofol	25 (%16.0)	8 (%6.4)	
**Complications**			
Hypertension	22 (%14.1)	8 (%6.5)	**0.040**
Hypotension	1 (%0.6)	-	-
Desaturation Allergic Rash Respiratory depression	10 (%6.4) 1 (%0.6) 1(%0.6)	5 (%4.0) - 1(%0.6)	0.380 - -

EBUS-TBNA: Endobronchial ultrasound-guided transbronchial needle aspiration. Data are expressed as n (%), median (min-max). p < 0.05: Significant statistically difference among group

Although there was also a statistically significant difference in terms of the distribution of anesthetic agents between the age groups (p < 0.05) (Table 2), the propofol-ketamine combination was the most preferred sedative in both groups. Propofol alone was the least preferred in Group 2 (p < 0.05) (Table 2). Propofol-ketamine and propofol-ketamine-midazolam were used more frequently in the 45 and younger age group. In comparison, propofol-ketamine and propofol-midazolam were used more frequently in the 65 and older age group (Table 2). The propofol-ketamine combination was the most preferred drug regimen in both groups. The usage rate of the propofol-ketamine combination was 37.8% and 49.2% in Groups 1 and 2, respectively) (Table 2).

Drug doses used in propofol-midazolam, propofol-ketamine, propofol-ketamine-midazolam, and only propofol regimens were compared between the groups. The dose of propofol used in the regimen in which propofol was administered alone was found to be statistically significantly lower in the elderly group (p < 0.05) (Table 3). No significant difference was found for the doses of each drug in the other drug regimens (p > 0.05) (Table 3).

The changes in MBP, HR, and SpO_2_ values according to the basal value (T1) were compared between the groups. The increase in the HR was statistically significant in Group 2 in the T4 and T5 periods with respect to T1 when the differences were compared (p < 0.05) (Table 4). When the MBP and SpO_2_ differences between baseline values (T1) and the values in T2, T3, T4, and T5 time points were compared, no significant difference was found between the groups (p > 0.05) (Table 4).

**Table 3:** Distribution of drug regimens by Groups

Drug regimens		Group 1 ≥ 65 Age (n:156)	Group 2 ≤ 45 Age (n:124)	p-value
Propofol-Midazolam (n:72)	Propofol	80(30–180)	100(30–350)	0.116
	Midazolam	2(1–5)	2(1–10)	0.187
Propofol-Ketamine (n:120)	Propofol	50(15–130)	50(20–220)	0.256
	Ketamine	45(10–130)	50(20–135)	0.106
Propofol-Ketamine Midazolam (n:55)	Propofol	42.5(20–100)	50(20–190)	0.117
	Ketamine	40(20–100)	45(20–120)	0.658
	Midazolam	2(1–3)	2(1-3.5)	0.672
Propofol (n:33)		80(30–220)	115(60–240)	**0.046**

Data are expressed as median (min–max). p < 0.05: Significant statistically difference among group

**Table 4:** The differences of the values of the hemodynamic parameters and SpO_2_ according to the time periods

Parameters	Group 1 ≥ 65 Age (n:156)	Group 2 ≤ 45 Age (n:124)	p-value
**MPB (mmHg)**			
T2- T1	0.41 ± 8.62	1.19 ± 7.11	0.163
T3- T1	1.28 ± 12.29	2.70 ± 10.19	0.139
T4- T1	1.28 ± 13.38	2.73 ± 9.42	0.285
T5- T1	0.90 ± 13.65	2.99 ± 11.47	0.354
**HR (beat/min)**			
T2- T1	0.96 ± 8.33	2.71 ± 8.30	0.198
T3- T1	1.10 ± 8.36	3.77 ± 9.28	0.056
T4- T1	1.15 ± 8.72	4.18 ± 1.14	**0.012**
T5- T1	1.56 ± 9.77	4.83 ± 11.22	**0.017**
**SpO**_**2**_ **(%)**			
T2- T1	-0.29 ± 4.28	0.04 ± 1.86	0.856
T3- T1	-0.41 ± 3.44	-0.40 ± 1.92	0.535
T4- T1	-0.35 ± 2.63	-0.39 ± 2.01	0.870
T5- T1	-0.09 ± 2.42	-0.66 ± 3.43	0.575

MBP: Mean blood pressure. HR: Hearth rate. SpO_2_: Peripheral oxygen saturation

T1: Before sedation, T2: After induction, T3: After the ultrasonic bronchoscope passed the vocal cords, T4, T5: Every 3 min during the procedure

Data are expressed as mean ± SD. p < 0.05: Significant statistically difference among group

The frequency of high blood pressure as a complication was found to be higher in Group 1, with a rate of 14.1% (p < 0.05) (Table 5). Although it was not statistically significant, the rate of hypertension (20.3%) in the propofol-ketamine group was found to be higher in the elderly group. The hypertension rate was higher in the propofol group (12.5%) in the younger group (p > 0.05) (Table 5).

**Table 5:** Distribution of hypertensive patients by drug regimens

Drug regimens	Hypertensive patient		p-value
	Group 1	Group 2	
Propofol-Midazolam	(n:46) 3(%6.5)	(n:26 ) -	0.549
Propofol-Ketamine	(n:59) 12(%20.3)	(n:61) 5(%8.2)	0.062
Propofol-Ketamine Midazolam	(n:26) 4(%15.4)	(n:29) 2(%6.9)	0.406
Propofol	(n:25) 3(%12)	(n:8) 1(%12.5)	0.999

Data are expressed as n (%). p < 0.05: Significant statistically difference among group

One patient in each group developed respiratory depression and required intubation as a major complication. The patient who was intubated in Group 1 was a 70-year-old female who received 3 mg of midazolam, 20 mg of ketamine, and 20 mg of propofol during the procedure. The patient who was intubated in Group 2 was a 35-year-old female who received 2 mg of midazolam, 40 mg of induction, and 160 mg of maintenance propofol during the procedure. Both patients were extubated shortly after the procedure without any delay in the procedure or recovery times. There was no statistically significant difference between the groups regarding desaturation frequency (p > 0.05). Desaturation occurred in 6.4% of patients in the elderly group and 4% in the younger group (Table 2). Low blood pressure and allergic rash occurred in one patient in the elderly group.

## Discussion

In this study, our DS applications during EBUS-TBNA were safe in both geriatric and young patients. Although PFTs values were found to be lower in the elderly group before the procedure, SpO_2_ values and desaturation rates were statistically similar in both groups during the procedure. The procedure was completed with similar recovery times in both groups, despite a higher ASA score and greater accompanying comorbidities in the elderly group. When the sedative approach we used in EBUS-TBNA was evaluated, it was seen that the propofol-ketamine combination was mostly preferred in both age groups. A significant decrease in propofol doses was observed in elderly patients who were administered propofol only. In terms of complications, except for an increase in blood pressure in elderly patients, complications were similar in both groups.

The ideal sedation protocol for EBUS-TBNA has yet to be determined. In the literature, midazolam is frequently used as a sedative agent in minimal sedation, while fentanyl and midazolam are combined for moderate sedation [11, 24, 25]. Midazolam is the most commonly preferred benzodiazepine due to its rapid onset of action, short duration, and reversible properties [26].

In DS, patients’ consciousness level is suppressed to a level where they will not wake up quickly but will respond to repeated or painful stimuli [3, 8]. Respiratory function should be maintained, but there is a risk of insufficient spontaneous ventilation. In the studies using DS without the use of an artificial airway, in which spontaneous respiration was maintained, propofol-midazolam [27], propofol-ketamine [28], and combinations of propofol, ketamine, midazolam, and fentanyl [25, 29] were safely used. In our study, we safely used different combinations of propofol, midazolam, and ketamine as sedative agents. Propofol is the most commonly used rapid-acting sedative agent with an acceptable safety profile in DS. It also provides quick recovery but has no analgesic effect. It has both antiemetic and amnestic effects. There is a risk of respiratory depression and hypotension at high doses [12]. Anesthesia depth can be achieved by combining it with other drugs, and the procedure time can be prolonged. It is recommended to be used by anesthesiologists in bronchoscopic procedures [30]. Respiratory depression is one of the most severe complications during sedation. We had one patient in each group who developed respiratory depression and had to be intubated. Both patients completed the procedure and were extubated without complication. The elderly patient was sedated with propofol, ketamine, and midazolam, and the young patient with propofol and midazolam. Respiratory depression had occurred in the young patient after propofol administration due to worse procedural tolerance. Good procedural tolerance in elderly patients who underwent EBUS-TBNA has also been reported in previous studies [9, 23]. We thought that dose reduction should be considered when sedation is applied with multiple anesthetic agents in elderly patients.

Ketamine provides rapid, deep sedation and analgesia. When administered slowly, it preserves respiratory and airway reflexes. It slightly increases HR and blood pressure and causes bronchodilation. It is a good choice for sedation in patients with airway sensitivity. Adding ketamine to sedation with midazolam or propofol can reduce hypoventilation and dose-dependent side effects [28, 29, 31, 32].

There are limited studies evaluating the effectiveness and safety of EBUS-TBNA under sedation in elderly patients. Evison et al. [23] found that elderly patients over 70 had poor performance and required lower sedation doses in a prospective cohort study. In their study, conscious sedation was applied with midazolam and alfentanil, and the complication rates in the elderly group were similar to those in the younger group, except for two significant complications. The younger group showed worse procedural tolerance despite receiving higher sedation doses. There was no difference between the groups regarding procedure times in their study.

Niwa et al. [33] administered midazolam and fentanyl to patients aged 80 years and over who underwent EBUS-TBNA. They reported a complication rate of 5%, including excessive sedation, chest pain, arrhythmia, and hypoxia. They reported that excessive sedation developed in a patient who was given midazolam 3 mg.

Okachi et al. [21] found that elderly patients over 70 years of age who underwent light sedation with midazolam were similar to the younger group in terms of systolic blood pressure increase during the procedure although their baseline systolic blood pressure values were high. They did not find any statistical difference in terms of minimum SpO_2_ level, maximum oxygen support, and HR during the procedure. In our study, baseline MBP values were higher in the elderly group. The fact that the MBP change according to the baseline value was similar in both groups during the procedure suggested that the sedation applied was safe. The fact that the incidence of hypertension was higher in the elderly group in combinations in which ketamine was used made us think that this group is more sensitive to the effect of ketamine.

Comorbidities such as heart, lung disease, DM, and renal pathology in geriatric patients pose a risk for postoperative mortality [34, 35]. The incidence of comorbidities such as DM, CO8PD, CAD, and ASA risk scores were significantly higher in our elderly group. In 75% of surgical patients over 70 years of age, the presence of one or more accompanying health problems was noted, with hypertension (46.6%) being the most common problem [36]. Similarly, hypertension was the most common underlying disease (43.6%) in our elderly group. Cerit et al. [37] reported that comorbidities were the leading cause of possible complications in geriatric patients. They reported that selecting the appropriate anesthesia method and providing adequate monitoring could reduce anesthesia complications. Although comorbidities were more common in the elderly group, we found similar results regarding desaturation, hypotension, and recovery time in the younger group. We believe that the minimal invasiveness of the EBUS-TBNA procedure, pre-anesthesia examination [34], the selected anesthetic agents, and adequate monitoring reduced these risks despite increased comorbidities in our older patients. Unlike the study conducted by Eapen et al. [17], we did not observe an increase in the level of care in patients aged 65 and above who underwent EBUS-TBNA under DS in our study. Compared to the younger group, there was no significant difference in complications except for transient high blood pressure, which was controlled with nitroglycerin. Despite studies [8, 11, 27] demonstrating that the type of anesthesia has no effect on the diagnostic yield of EBUS-TBNA, there are also studies showing the opposite. The studies have shown that DS or general anesthesia provides greater patient comfort, more lymph node station sampling ability, higher diagnostic yields [10, 13, 18], and shorter procedure times [10]. There may be no difference in sedation type for uncomplicated cases and experienced practitioners. However, for practitioners who do not routinely perform the procedure, DS may increase the safety and performance of the procedure [12, 13]. In many studies that used DS, artificial airways (laryngeal mask or endotracheal tube) were used to provide ventilation [8, 10, 13, 38]. The number of studies that have safely and effectively performed the procedure without the use of artificial airways under DS in the general population is limited [25, 27, 28]. In our study, we performed procedures under DS without the need for artificial airways.

Demirci et al. [39] reported no complications in 96.6% of elderly patients aged 65 years and older who underwent EBUS-TBNA using midazolam for sedation. The complications in the elderly group included hemorrhage controlled with cold saline, mild respiratory depression requiring high-flow oxygen, transient tachycardia, and fever in their retrospective study. In another retrospective observational study by Yıldızeli et al. [22], they reported a complication rate of 7.7%, including hypoxia, fever, and tachycardia in patients aged 70 and older who received midazolam.

Dhooria et al. [9] used conscious sedation with midazolam, pentazocine, and fentanyl in patients aged 65 and over. They reported sustained hypoxemia, bleeding, and excessive coughing as the most common complications. They observed a significant increase in heart rate during the procedure in the younger group. We also observed a higher increase in heart rate in the younger group. The dose of midazolam was found to be higher in their young group. Interestingly, although not statistically significant, there were more patients with increased care levels in that group.

Increased body fat, decreased total body water, liver volume, blood flow, hepatic enzyme activity, and renal clearance in elderly patients can lead to prolonged drug effects. It is generally recommended to reduce the dose of agents used in anesthesia in elderly patients. Opioid requirements are also reduced in these patients [36]. Previous studies have also reported lower sedation doses in the elderly group [21, 23]. A significant decrease in propofol doses was observed in elderly patients who were administered propofol only. In our study, we thought that propofol doses were cautiously administered to avoid complications in elderly patients using propofol alone.

All of these limited studies conducted in elderly patients have shown that EBUS-TBNA with conscious sedation is a safe and well-tolerated procedure despite advanced age and comorbidities. Effective minimal invasive procedure for accurate nodal staging and pathological confirmation has been shown not to increase the risk of complications associated with age [9, 21, 22, 33, 39]. Our study also demonstrated that EBUS-TBNA can be safely performed in elderly patients under DS.

There are some limitations in our study. First, the study was performed retrospectively in a single center. Although the sedative agent doses used in this study have been revealed, prospective studies with specific drug doses in elderly patients are needed. Secondly, because of the fact that the EBUS-TBNA procedures were performed in the high-volume tertiary pulmonology center by experienced pulmonologists and anesthesiologists may have decreased the complications, our results may not reflect the general patient population. Finally, a variety of sedative agents and several combinations in different doses were used in the study. This was attributed to the sedation practice of different anesthesiologists in the interventional pulmonology department.

## Conclusion

The EBUS-TBNA procedure performed under DS was safe in both elderly and young patients. In elderly patients, the increase in blood pressure observed during the procedure responded promptly to treatment and did not create any risk. Our study showed that the procedure and recovery times under DS were similar in elderly and young groups. We found that the choice of the sedative agent of the anesthesiologist changed according to age. At the same time, we observed that lower doses of propofol were preferred in the elderly group receiving only propofol. This has shown us that anesthetists are more sensitive to the application of sedative agents in geriatric patients, considering comorbidities and drug interactions related to multi-medication.

## Data Availability

The data sets analysed during the this study are available from the corresponding author on reasonable request.

## Abbreviations

EBUS-TBNA: Endobronchial ultrasound-guided transbronchial needle aspiration
DS: Deep sedation
PFTs: Pulmonary function tests
SpO_2_: Peripheral oxygen saturation
FOB: Fiberoptic bronchoscope
ASA: American Society of Anesthesiologists
BMI: Body mass index
DM: Diabetes mellitus
HT: Hypertension
CAD: Coronary artery disease
COPD: Chronic obstructive pulmonary disease
MBP: Mean blood pressure
SBP: Systolic blood pressure
HR: Heart rate
RSS: Ramsey Sedation Scale
